# Shikonin is a novel antagonist of prostaglandin E2 receptor 4 that targets myeloid-derived suppressor cells

**DOI:** 10.1016/j.gendis.2024.101356

**Published:** 2024-06-20

**Authors:** Yang Wang, Naijipu Abuduaini, Wenjuan Liu, Yuanjun Song, Zunping Ke, Xilong Wang, Wei Jiao, Si Chen, Xianhua Lin, Weiwei Yu, Weiqiang Lu, Bo Feng, Jiacheng He

**Affiliations:** a Department of Urology, The Fifth People's Hospital of Shanghai, Fudan University, Shanghai 200240, China; b Department of General Surgery, Ruijin Hospital, Shanghai Jiao Tong University School of Medicine, Shanghai 200020, China; c Shanghai Key Laboratory of Regulatory Biology, Institute of Biomedical Sciences and School of Life Sciences, East China Normal University, Shanghai 200241, China; d Shanghai Municipal Health Commission, Shanghai 200125, China; e Department of Gerontology, The Fifth People's Hospital of Shanghai, Fudan University, Shanghai 200240, China; f Joint Center for Translational Medicine, Shanghai Fifth People's Hospital, Fudan University and School of Life Science, East China Normal University, Shanghai 200240, China

Myeloid-derived suppressor cells (MDSCs) constitute a crucial component of the immunosuppressive tumor microenvironment.^1^ Prostaglandin E2 receptor 4 (EP4) is involved in regulating immunosuppressive MDSC differentiation and is emerging as a promising target for cancer immunotherapy.^2^ No EP4 antagonists have been approved for anti-tumor therapy, underscoring the urgent requirement for the discovery of novel EP4 antagonists. G protein and β-arrestin represent two classical downstream pathways for EP4. The inactivity of G protein and β-arrestin serves as a readout to indicate EP4 antagonism, providing a rationale for establishing EP4 drug screening platforms. From a broad perspective on the history of G protein-coupled receptor (GPCR) drug discovery, using a β-arrestin-based drug screening strategy may offer greater advantages over G protein strategies, especially for the GPCRs that have not been proven on which G proteins they bind. Several cellular assays for the detection of GPCR/β-arrestin interaction have been established, including the PRESTO-Tango assay and fluorescent β-arrestin labeling assay. However, these assays are not suitable for the real-time dynamic detection of GPCR-β-arrestin signaling. In this study, we aimed to develop a novel real-time β-arrestin recruitment assay for EP4 receptor and to identify a potent EP4 antagonist that could attenuate the immunosuppressive effects of MDSCs.

To accelerate the identification of EP4 antagonists, we developed a nanoluc luciferase-based real-time β-arrestin recruitment (RTAR) assay (Fig. 1A). Nanoluc luciferase was artificially divided into two fragments, large Bit (LgBit, 17.6 kDa) and small Bit (SmBit, 11 amino acids). EP4 receptor was genetically fused to the LgBit or SmBit fragment at the C-terminal, and the remaining fragment was fused to the N- or C-terminal of β-arrestin, resulting in four different constructs pairs (Fig. S1A). As shown in Figure S1B–E, endogenous agonist PGE2 (10 nM) effectively elevated the bioluminescence in RTAR assay, demonstrating the physical complementation of the nanoluc fragments driven by EP4/β-arrestin interaction. Moreover, the maximal response of PGE2 was achieved on EP4-LgBit and SmBit-β-arrestin combination with a ratio of 1:1, exhibiting an approximately 2.5-fold increase of bioluminescence when compared with the control group (Fig. S1B). Notably, PGE2 failed to increase bioluminescence in the absence of EP4 or β-arrestin genes of the constructs (Fig. S1F).

**Figure 1:**
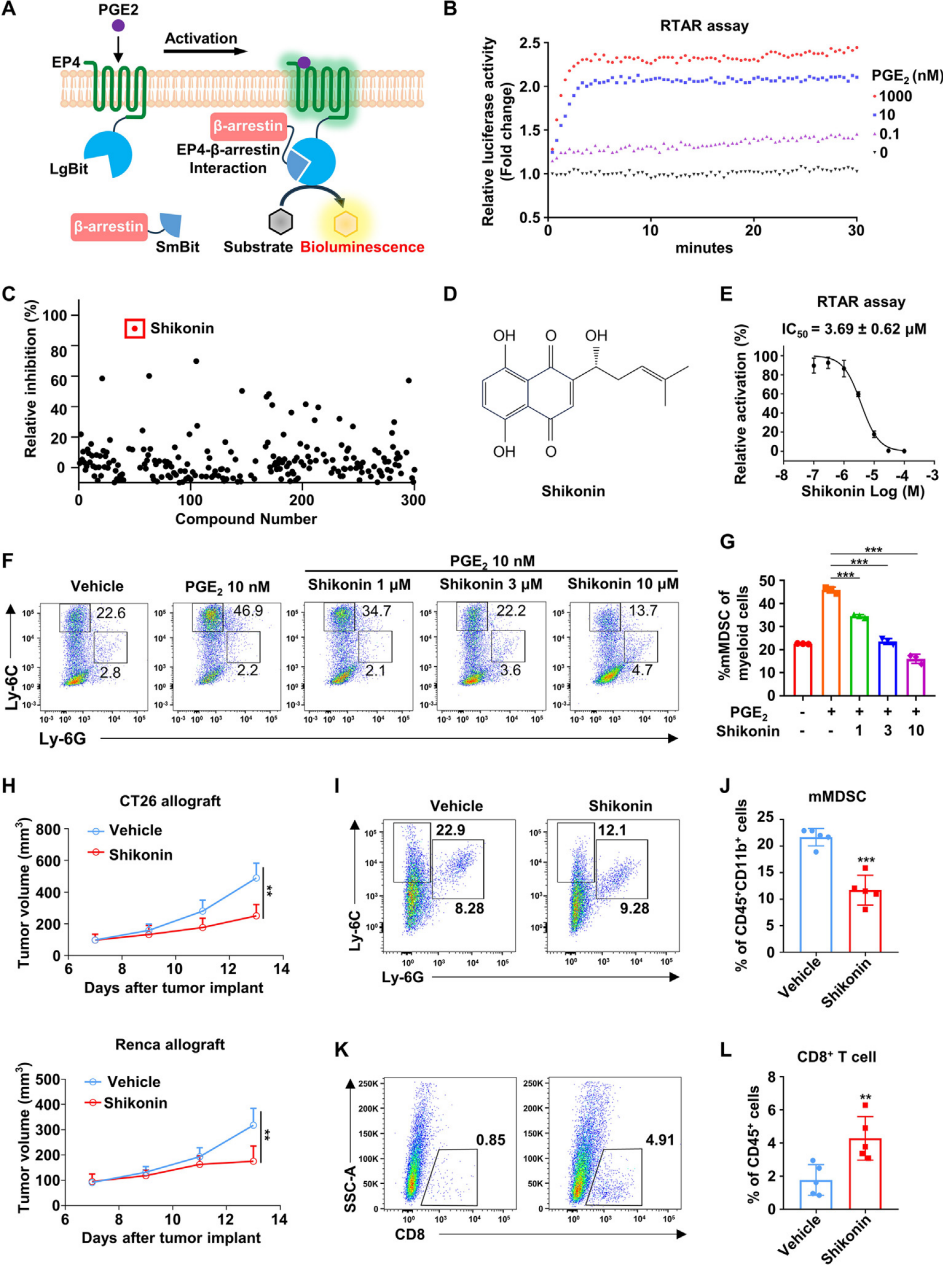
Shikonin is a novel antagonist of prostaglandin E2 receptor 4 (EP4) that targets myeloid-derived suppressor cells (MDSCs). **(A)** Schematic illustration of the RTAR assay. The nanoluc luciferase complementation system consists of two fragments, large Bit (LgBit, 17.6 kDa) and small Bit (SmBit, 11 amino acids). EP4 receptor and β-arrestin are fused with different fragments of nanoluc, LgBit, and SmBit, respectively. EP4 activation by agonist recruits the β-arrestin to the cell membrane. The spatial reassembling of nanoluc luciferase occurs when EP4 interacts with β-arrestin, which results in strong nanoluc luciferase activity. **(B)** RTAR assay monitoring curves of PGE2-induced EP4 receptor activation. The concentration of PGE2 is indicated in figure. **(C)** Inhibitory rates of the compounds from a natural product library on EP4 at 10 μM in the RTAR assay. Each dot represents one compound. **(D)** The chemical structure of Shikonin. **(E)** Dose–response curve of Shikonin on EP4 receptor in RTAR assay. 10 nM PGE2 was used (*n* = 3). **(F, G)** Representative flow cytometry dot plots (F) and corresponding quantification (G) of the frequencies of monocytic MDSCs (mMDSCs; CD11b^+^Ly-6C^hi^Ly-6G^−^) in marrow-derived cells treated with 40 ng/mL interleukin-6, 40 ng/mL granulocyte macrophage-colony stimulating factor, 10 nM PGE2, and/or indicated concentrations of Shikonin (*n* = 3). **(H)** Tumor growth curves of CT26 allograft-bearing (upper panel) and Renca allograft-bearing (lower panel) BALB/c mice treated with 5 mg/kg Shikonin or vehicle control (*n* = 5). **(I, J)** Representative flow cytometry dot plots (I) and corresponding quantification (J) of the frequencies of mMDSC (Ly-6C^hi^Ly-6G^−^) gated on CD45^+^ CD11b^+^ cells in the CT26 allograft tissues (*n* = 5). **(K, L)** Representative flow cytometry dot plots (F) and corresponding quantification (G) of the frequencies of CD8^+^ cells gated on CD45^+^ cells in the CT26 allograft tissues (*n* = 5). All data were represented as mean ± standard deviation. Two-tailed unpaired student's *t*-tests were performed to compare data between two groups. One-way ANOVA with Tukey's multiple comparison tests were performed to compare data among three or more groups. ∗∗*P* < 0.01, ∗∗∗*P* < 0.001.

Then, we turned to determine the potency of several well-known EP4 ligands by our RTAR system. As shown in Figure 1B, PGE2 could elicit a dose-dependent increase of bioluminescence in the range of 0.1 nM–1000 nM, and the luminescence signal was stable for at least 30 min (Fig. 1B). PGE2 had an EC_50_ value of 1.15 ± 0.24 nM (Fig. S2A) in the RTAR assay, which was highly consistent with previously reported data which was highly consistent with previously reported data.^3^ CJ-042794, a well-known EP4 antagonist, displayed a potent inhibition on PGE2-induced bioluminescence increase with an IC_50_ value of 24.01 ± 4.32 nM (Fig. S2B), which was consistent with previous studies.^4^ Collectively, these data suggest that the RTAR assay is suitable for the real-time assessment of EP4 agonism and antagonism.

To identify novel EP4 antagonists, we performed the new-developed RTAR assay by screening a natural product library with diverse structural skeletons. Among 300 natural products, Shikonin exhibited the highest antagonistic effect on the EP4 receptor at a concentration of 10 μM (Fig. 1C, D). Shikonin, a natural product from the roots of *Lithospermum erythrorhizon*, is a traditional Chinese medicinal herb. Specifically, Shikonin treatment resulted in a dose-dependent reduction of EP4/β-arrestin interaction aroused by PGE2 with an IC_50_ value of 3.69 ± 0.62 μM in the RTAR assay (Fig. 1E). EP4 was reported to couple to Gαs protein and increase intracellular second messenger cAMP. To further assess the antagonism effect of Shikonin on EP4 receptor, we performed a cAMP Glosensor assay using a genetically engineered luciferase cAMP biosensor. Shikonin dose-dependently inhibited PGE2-induced cAMP accumulation with an IC_50_ value of 4.11 ± 0.36 μM, consistent with the result of the RTAR assay (Fig. S2C). Furthermore, a similar antagonistic activity of Shikonin was also observed by EP4 Ca^2+^ flux assay with an IC_50_ value of 3.48 ± 0.38 μM (Fig. S2D). To reveal the interaction model of EP4 and Shikonin, we performed molecular docking using Autodock. Shikonin exhibited a robust binding affinity to the EP4 core pocket, with residues including V72, T76, Y80, W85, L99, T168, W169, L312, I315, R316, and S319 (Fig. S2E, F). Among these, residues T76, S319, and R316 were identified as pivotal amino acids to form hydrogen bonds for the establishment of a strong interaction with Shikonin (Fig. S2E, F). Taken together, these data suggest that Shikonin is a natural EP4 antagonist with a unique chemical skeleton.

MDSCs are one of the primary components in the immunosuppressive tumor microenvironment. Historically, the increased expression of arginase 1 (ARG1) in MDSCs is responsible for the immunosuppressive effect. Our previous studies have demonstrated that EP4 is highly expressed in MDSCs and inhibition of EP4 significantly suppresses the differentiation of monocytic MDSCs.^5^ To validate the functional role of EP4 antagonist Shikonin in suppressing MDSC differentiation, we established an MDSC bone marrow differentiation model by incubating the bone marrow-derived cells with granulocyte macrophage-colony stimulating factor and interleukin-6. Fluorescence-activated cell sorting (FACS) analysis showed that exposure of marrow-derived cells to PGE2 significantly increased the population of monocytic MDSCs (Ly-6G^−^Ly-6C^hi^), and this effect could be abrogated by Shikonin (Fig. 1F, G). Additionally, Shikonin suppressed the expression of *Arg1* induced by PGE2 in the differentiated monocytic MDSCs in a dose-dependent manner (Fig. S3A). Collectively, our data indicate that Shikonin inhibits the differentiation of monocytic MDSCs.

To evaluate the *in vivo* anti-tumor potential of Shikonin, we established a syngeneic murine renal cancer model and a syngeneic murine colorectal cancer model using CT26 cells and Renca cells. CT26 allograft- or Renca allograft-bearing mice were administrated with 5 mg/kg Shikonin or vehicle control via intraperitoneal injection after the tumor volume reached approximately 100 mm^3^. Shikonin treatment significantly inhibited the growth of CT26 allograft and Renca allograft, with tumor growth inhibition (TGI) rates of 49% and 45%, respectively (Fig. 1H). To investigate the effects of Shikonin on intra-tumoral monocytic MDSC accumulation and the immunosuppressive function in the tumor microenvironment, we dissected the tumors and performed FACS analysis and real-time PCR analysis. As shown in Figure 1I, J and S3B, Shikonin treatment significantly inhibited the intra-tumoral accumulation of monocytic MDSCs, and suppressed the expression of the immunosuppressive enzyme *Arg1* compared with the vehicle-treated mice. Furthermore, Shikonin-treated mice exhibited a higher abundance of intra-tumoral cytotoxic CD8^+^ T cells than those in vehicle-treated mice (Fig. 1K, L). These data suggest that Shikonin attenuates tumor immunosuppression and inhibits tumor growth in both renal cancer and colorectal cancer.

In conclusion, we have developed a precise, real-time, live-cell, and broadly applicable RTAR assay for GPCR drug discovery. Using this assay, we identified a novel EP4 antagonist Shikonin. Inhibition of EP4 by Shikonin significantly suppressed MDSC differentiation and restrained the growth of colorectal cancer and renal cancer (Fig. S4). Our findings suggest that Shikonin represents a prospective strategy for cancer immunotherapy.

## Ethics declaration

All animal experiments were performed in accordance with the Guiding Principles for the Care and Use of Laboratory Animals. All murine experiments were approved by the Ethics Committee at East China Normal University.

## Funding

This work was supported by the Shanghai Key Medical Specialty Program (China) (No. ZK2019A03 to Y.W.), Natural Science Research Funds of Minhang District, Shanghai, China (No. 2021MHZ071 to Y.W.), Scientific Research Project funded by Shanghai Fifth People's Hospital, 10.13039/501100003347Fudan University (No. 2020WYZT02 to Y.W.), the 10.13039/501100001809National Natural Science Foundation of China (No. 82373237 to B.F., 82172644 to W.Q.L), ECNU Multifunctional Platform for Innovation (011), and The Instruments Sharing Platform of School of Life Science, East China Normal University.

## Conflict of interests

The authors declared no conflict of interests.
