# Supplementary material for: Shikonin is a novel antagonist of prostaglandin E2 receptor 4 that targets myeloid-derived suppressor cells
Source: Genes Dis. 2024 Jun 20;12(3):101356. doi: 10.1016/j.gendis.2024.101356 (PMC11803219; doi:10.1016/j.gendis.2024.101356)
Supplement: Multimedia component 1 [file mmc1.docx]

**Supplemental Material**

**Materials and methods**

**Cell culture**

HEK293, CHO-K1, CT26 and Renca cell lines were obtained from the American Type Culture Collection (Manassas, VA, USA). HEK293 and CT26 cells were cultured in Dulbecco's Modified Eagle Medium (DMEM, Gibco, USA). CHO-K1 cells were cultured in DMEM-Ham's F12 (DMEM/F12, Gibco). Renca cells were cultured in RPMI 1640 Medium (Gibco). All culture medium were supplemented with 10% Fetal bovine serum (Gibco), 100 U/mL penicillin and 100 mg/mL streptomycin (Gibco). Cells were maintained in a humidified incubator with 5% CO_2_ at 37°C. All cell lines were confirmed to be negative for mycoplasma by polymerase chain reaction (PCR).

**Mice**

BALB/c mice were obtained from Shanghai SLAC Laboratory Animal Co., Ltd (China). All murine experiments were approved by the Ethics Committee at East China Normal University. Mice were raised in the East China Normal University under specific pathogen-free conditions with temperature maintained at 20-22 °C, humidity at approximately 60%, and a 12-hour light/dark cycle. All mice were treated according to the Guide for the Care and Use of Laboratory Animals.

**Syngeneic murine tumor models**

Syngeneic murine tumor models were established by subcutaneous inoculation of 1,000,000 Renca cells (murine renal cancer cell) or CT26 cells (murine colorectal cancer cell) into the back of 8-week-old female BALB/c mice. Mice were anesthetized with isoflurane during tumor implantation. Once the tumor volume reached approximately 100 mm³, the mice were randomly assigned to two groups for treatment: one group received vehicle control, and the other group received 5 mg/kg of Shikonin (Sigma, USA) via intraperitoneal injection twice a week. The administration method for Shikonin was selected based on previous published studies^1;2^. Tumor volume and body weight were measured every two days. Tumor volume was calculated as 0.5 × length × width × width. Tumor volumes reaching approximately 400 mm^3^ were considered as the primary endpoints. At the end of experiment, mice were euthanized. Tumors were dissected to generate single-cell suspensions for FACS (fluorescence activated cell sorting) analysis and were homogenized for real-time PCR analysis.

**Real-time β-arrestin recruitment (RTAR) assay**

The RTAR assay was constructed with the fusion proteins composing of EP4 receptor and β-arrestin via a linker with the individual fragments of Nanoluc luciferase (LgBit and SmBit). The cDNA plasmids of EP4 and β-arrestin were purchased from YouBio (China).

HEK293 cells were transfected with four different set-ups in RTAR assay using Lipofectamine 2000 reagent (Thermo, USA) according to the manufacturer’s protocol. 20,000 transfected cells were seeded into 96-well plates (Costar, USA) and incubated overnight. After starvation for 2 hours in CO_2_-independent medium, cells were loaded with 2 μM furimazine (TargetMol, China) at room temperature for 30 min. Then, cells were treated with indicated concentration of PGE2 (Sigma, USA) and the luminescence was continuously measured by using Cytation 5 (BioTek, USA) for 30 min. In the case of antagonist evaluation, cells were pretreated with compounds for 10 min before PGE2 addition.

**Calcium flux assay**

EP4 calcium flux assay was performed as described previously ^3;4^. Briefly, CHO-K1 cells were transfected with EP4 and GNAQ genes for 6 hours. Cells were then seed into a 96-well microplate at a density of 20,000 cell per well. On the next day, cells were subjected to the reagent of Fluo-4 direct calcium assay kit (Thermo, USA) and exposed to the indicated compounds. Calcium flux was determined by the FlexStation 3 multi-mode microplate reader (Molecular Devices, USA).

**cAMP Glosensor assay**EP4 Glosensor cAMP assay was conducted as described ^3;4^. Briefly, HEK293 cells were transfected with an EP4 overexpressing plasmid (YouBio, China) and a GloSensor™-22F cAMP plasmid (Promega, USA) for 6 hours. On the next day, cells were seeded into a 384-well plate at a density of 20,000 cell per well. Subsequently, the cells were incubated with GloSensor™ cAMP reagent for 30 minutes according to the manufacturer's instructions. Cells were then exposed to the indicated compounds. Luminescence was measured by the FlexStation 3 multi-mode microplate reader.

***In vitro* MDSC differentiation**

Total bone marrow cells were flushed from femurs and tibias of 6-week-old female BALB/c mice with RPMI-1640 medium by using a syringe. The cell suspensions were filtered through 40 μm cell strainers (Millipore, Germany) and depleted of red blood cells with red blood cell lysis buffer. Then cells (1,000,000 cells/mL) were differentiated in the presence of 40 ng/mL GM-CSF and 40 ng/mL IL-6 at 37 °C (Day 1). On the third day, floating cells were gently removed and all the cell culture medium was updated. For FACS analysis, cells were treated with 10 nM PGE2 and/or indicated concentrations of Shikonin on day 1, followed by a 5-day incubation. For real-time PCR analysis, cells were treated with 10 nM PGE2 and/or indicated concentrations of Shikonin on day 5, followed by a 12-hour incubation.

**FACS analysis**

Singel-cell suspensions were prepared and washed with PBS containing 2 mM EDTA and 1% BSA (Sangon, China). Subsequently, cells were incubated with anti-CD16/32 FcR blocking antibody (Biolegend, USA, RRID: AB_1574973) for 10 minutes, followed by staining with antibodies at 4°C for 30 minutes. The cellular populations of mMDSC and PMN-MDSC were analyzed by using an LSR Fortessa flow cytometer (BD, USA). Antiboies used for FACS were listed as follows: APC anti-mouse Ly-6G Antibody (Biolegend, RRID: AB_1877163), PE anti-mouse Ly-6C Antibody (Biolegend, RRID: AB_1186132), APC anti-mouse CD8a Antibody (Biolegend, RRID: AB_312751), PerCP/Cy5.5 anti-mouse CD45 Antibody (Biolegend, RRID: AB_893340), FITC anti-mouse/human CD11b Antibody (Biolegend, RRID: AB_312788).

**RNA isolation and** **real-time PCR**

The RNA of the bone marrow-derived MDSCs or homogenized CT26 allografts was extracted using TRIzol reagent (Invitrogen, USA) in accordance with the instructions. The reverse transcription of mRNA into cDNA was performed by using PrimeScript™ RT Master Mix (Takara, Japan). The real-time PCR was conducted by Hieff^TM^ qPCR SYBR and Green Master Mix (Yesen, China). The primers of *Arg1* were listed as following: Forword (5’-3’) ACATTGGCTTGCGAGACGTA and Reverse (5’-3’) ATCACCTTGCCAATCCCCAG. β-actin was used as the internal reference.

**Data analysis**

For calculation of EC_50_ or IC_50_ values, a sigmoidal curve was fitted by analyzing data using GraphPad Prism 8.0. Relative activation %= [(RFU - R_min_)/ (R_max_ - R_min_)] × 100%, RFU refers to the relative fluorescence units measured in compounds-treated cells, R_min_ refers to the relative fluorescence units measured in vehicle-treated cells, and R_max_ refers to the relative fluorescence units measured in PGE2 treated cells. Two-tailed unpaired student’s t test was used to compare the difference of two groups, and One-way ANOVA with Tukey's multiple comparison test was used for comparison of multiple set of groups. P < 0.05 means statistic significant. Data were shown as the mean ± standard deviation. All the data analysis was carried out by GraphPad Prism 8.0. No data were excluded from analysis.

**Reference**

1. Wang F, Mayca Pozo F, Tian D, et al. Shikonin Inhibits Cancer Through P21 Upregulation and Apoptosis Induction. *Front Pharmacol.* 2020;11:861.

2. Liu FY, Wang MQ, Liu MM, et al. Therapeutic effects of shikonin on adjuvant-induced arthritis in rats and cellular inflammation, migration and invasion of rheumatoid fibroblast-like synoviocytes via blocking the activation of Wnt/beta-catenin pathway. *Phytomedicine.* 2023;116:154857.

3. Li W, Tanikawa T, Kryczek I, et al. Aerobic Glycolysis Controls Myeloid-Derived Suppressor Cells and Tumor Immunity via a Specific CEBPB Isoform in Triple-Negative Breast Cancer. *Cell Metab.* 2018;28(1):87-103 e106.

4. Lu W, Yu W, He J, et al. Reprogramming immunosuppressive myeloid cells facilitates immunotherapy for colorectal cancer. *EMBO Mol Med.* 2021;13(1):e12798.

**Supplementary Figures**


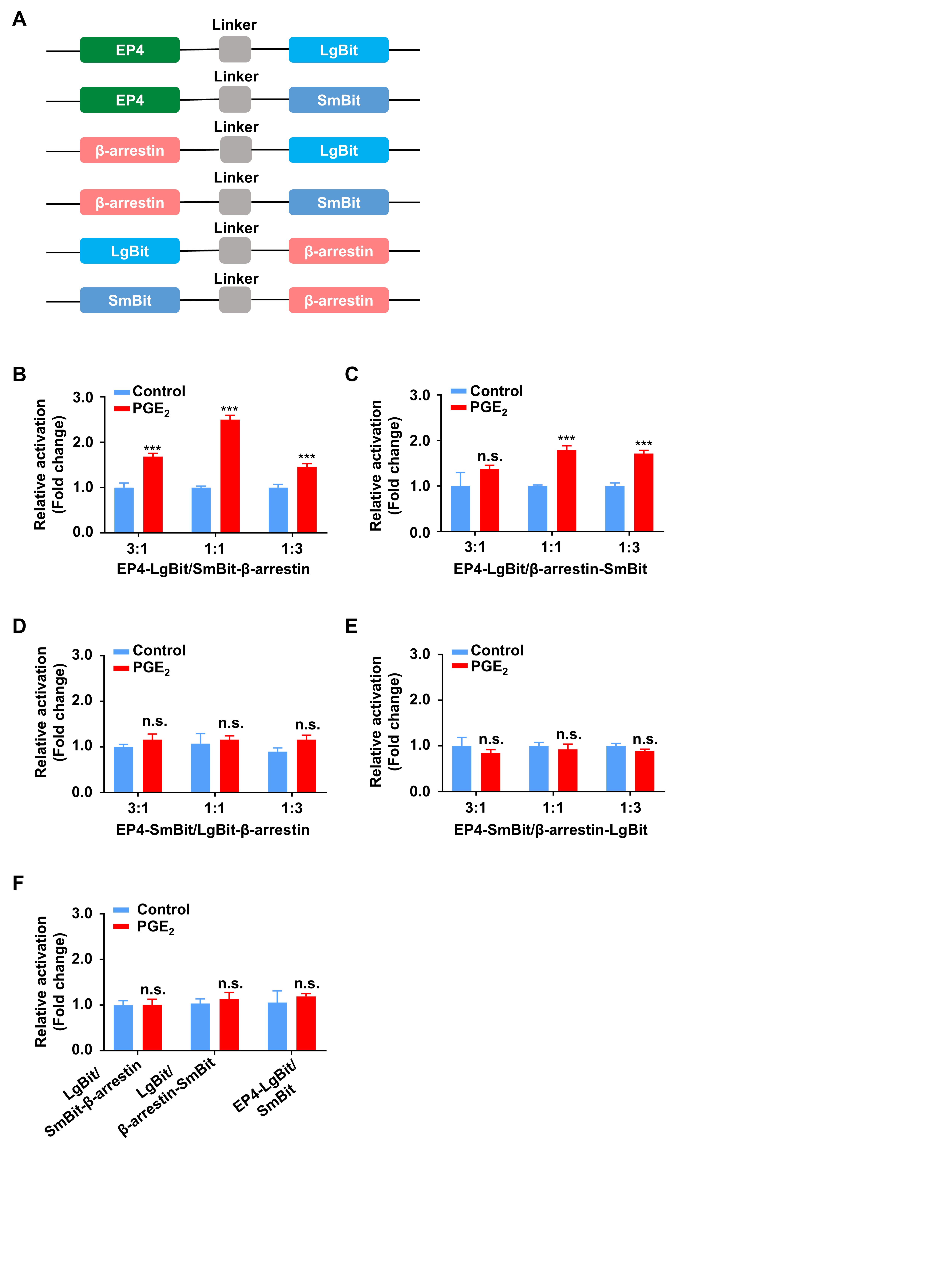
**Figure S1.** Optimization of the RTAR assay for EP4 receptor.

(A) Illustration of different constructs of RTAR assay system.

(B-E) The efficiency of four different combinations between EP4/β-arrestin and LgBit/SmBit. EP4-LgBit and SmBit-β-arrestin (B), or EP4-LgBit and β-arrestin-SmBit (C), or EP4-SmBit and LgBit-β-arrestin (D), or EP4-SmBit and β-arrestin-LgBit (E) were co-transfected into HEK293 cells with a ratio of 3:1, 1:1, 1:3. The transfected cells were treated with 10 nM PGE2 for 15 min, and the activity of bioluminescence was detected in the presence of substrate (n=3).

(F) LgBit/SmBit-β-arrestin, LgBit/β-arrestin-SmBit, and EP4-LgBit/SmBit were co-transfected into HEK293 cells with a ratio of 1:1. The transfected cells were treated with 10 nM PGE2 for 15 min, and the activity of bioluminescence was detected in the presence of substrate (n=3).

All data were represented as means ± standard deviation. Two-tailed unpaired student’s t tests were performed (***P<0.001; n.s., not significant).


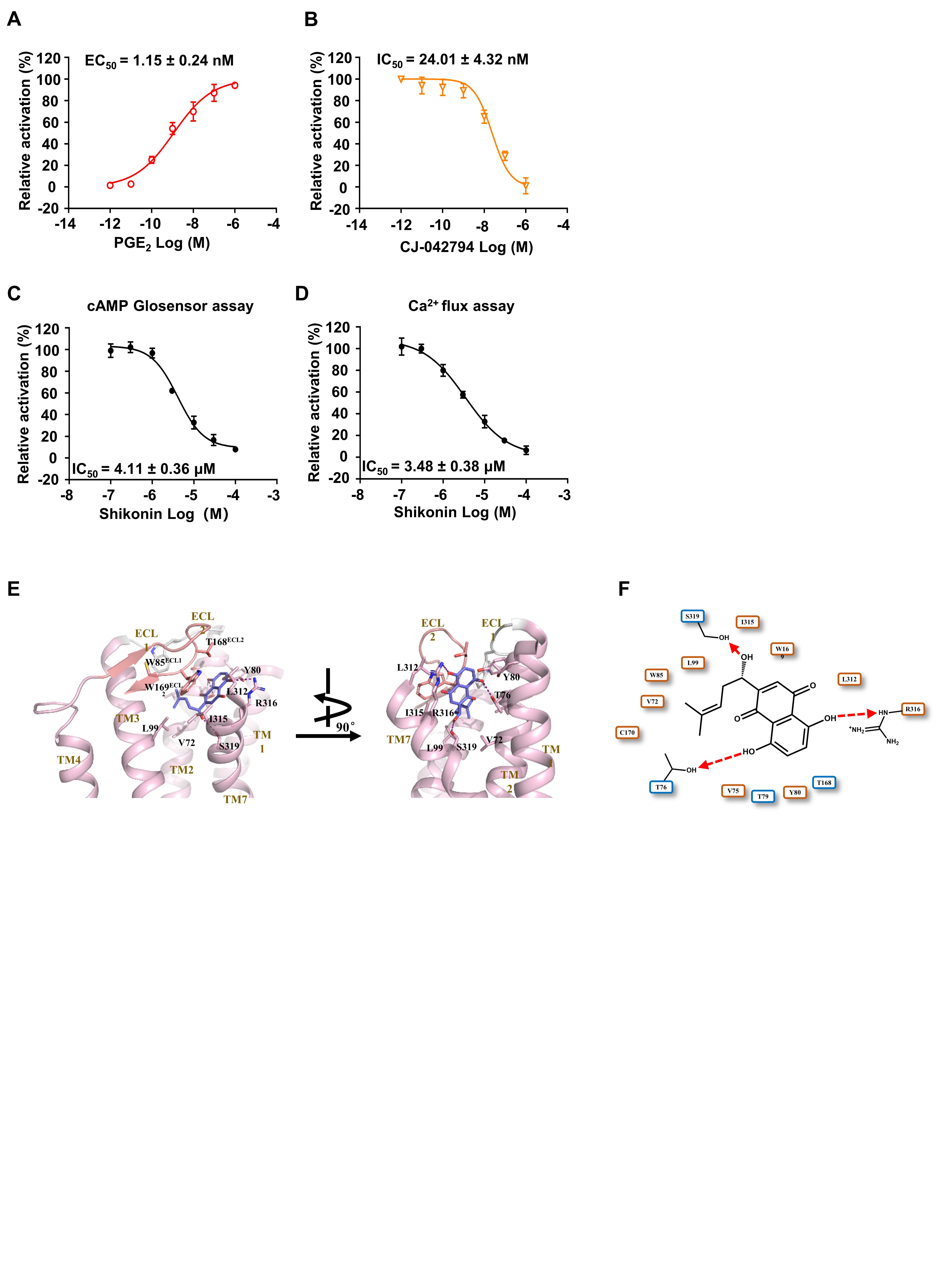
**Figure S2.** Screening and characterization of Shikonin as a novel EP4 antagonist.

(A) Dose-response curves of PGE2 on EP4 receptor in the RTAR assay (n=3).

(B) Dose-response curves of an EP4 antagonist CJ-042794 in the RTAR assay. 10 nM PGE2 (EC_80_) was used (n=3).

(C) Dose-response curves of Shikonin on EP4 receptor in cAMP Glosensor assay. 10 nM PGE2 was used (n=3).

(D) Dose-response inhibition curve of Shikonin on EP4 receptor in Ca^2+^ flux assay. 10 nM PGE2 was used (n=3).

(E) Top-scoring docking pose of Shikonin, shown as tv_blue sticks. Human EP4 is represented in light pink and the extracellular loops (ECLs) are represented in gray (ECL1) and salmon (ECL2) respectively. Side chain interactions within 4.0 Å residues are shown as sticks. Hydrogen bonds are shown as purple dashed lines. Human EP4 is rotated 90° around a vertical axis relative to A, Structures of TM3 and TM4 are removed for clarity.

(F) Diagram of ligand docking interactions between human EP4 and Shikonin. Hydrogen bonds are shown as red dashed lines. Residue names in brown and blue rectangle indicates hydrophobic and polar interaction with EP4, respectively.

All data were represented as means ± standard deviation.


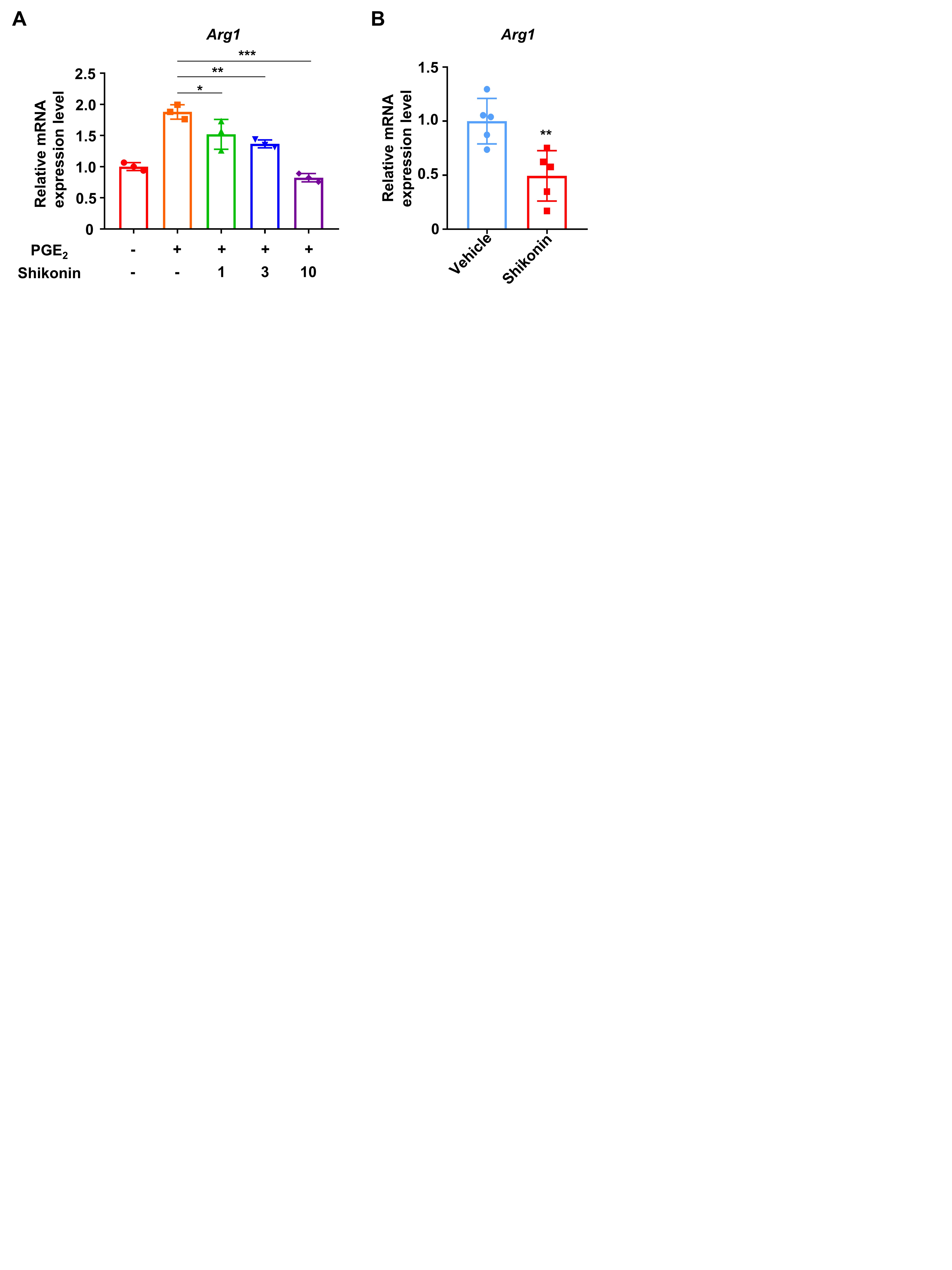


**Figure S3**. Shikonin suppresses the expression of *Arg1* in MDSCs.

(A) mRNA expression of *Arg1* in differentiated mMDSCs treated with 40 ng/ml IL-6, 40 ng/ml GM-CSF, 10 nM PGE2 and/or indicated concentrations of Shikonin (n=3).

(B) mRNA expression of *Arg1* in tumor tissues derived from CT26 allograft-bearing mice treated with 5 mg/kg Shikonin or vehicle control (n=5).

All data were represented as means ± standard deviation. One-way ANOVA with Tukey's multiple comparison tests were performed (A). Two-tailed unpaired student’s t tests were performed (B). (*P<0.05, **P<0.01, ***P<0.001).


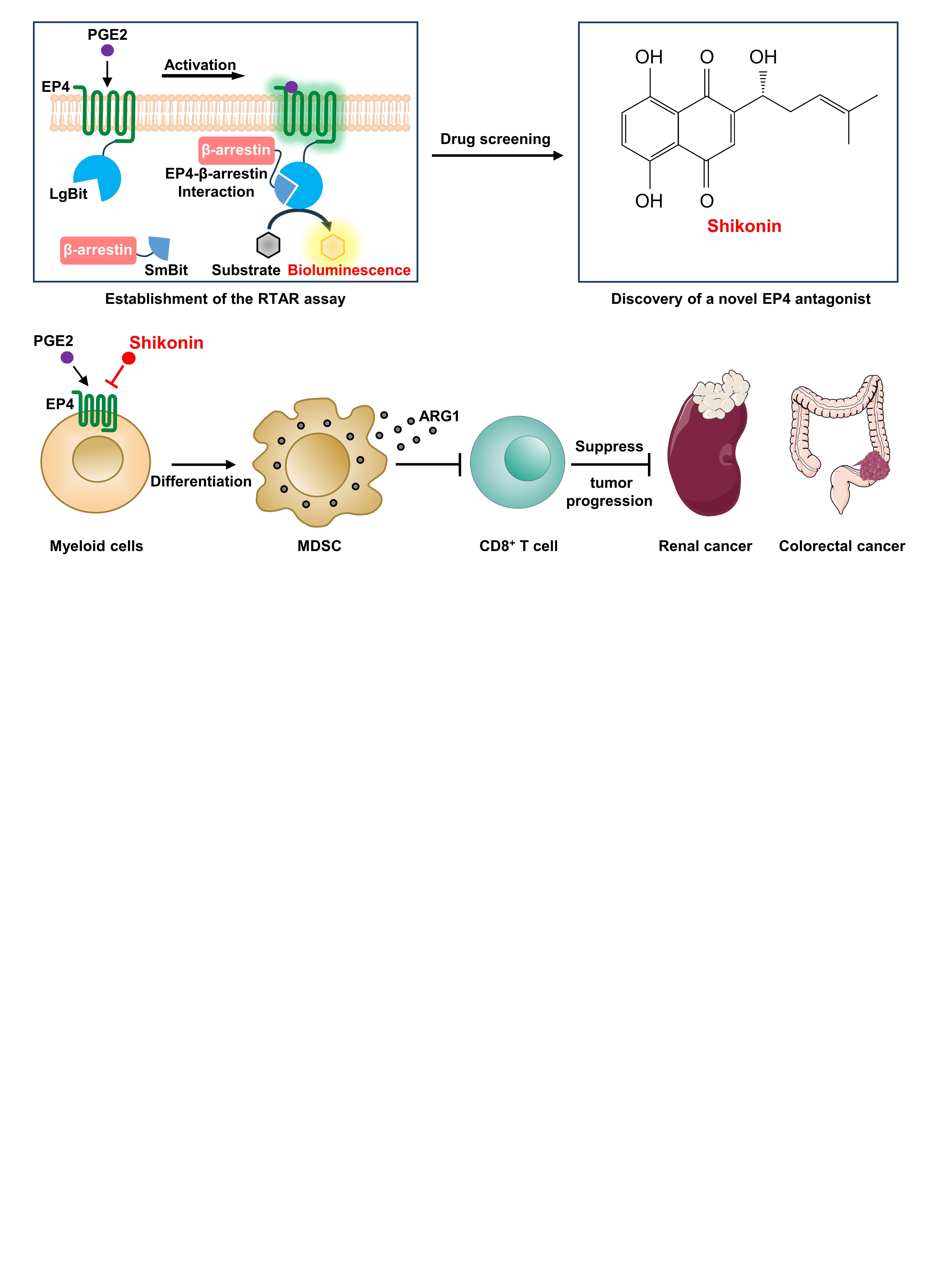


**Figure S4**. Graphical abstract of this study.
